# How to deal with uncertainty in prenatal genomics: A systematic review of guidelines and policies

**DOI:** 10.1111/cge.14010

**Published:** 2021-06-30

**Authors:** Jasmijn E. Klapwijk, Malgorzata I. Srebniak, Attie T. J. I. Go, Lutgarde C. P. Govaerts, Celine Lewis, Jennifer Hammond, Melissa Hill, Stina Lou, Ida Vogel, Kelly E. Ormond, Karin E. M. Diderich, Hennie T. Brüggenwirth, Sam R. Riedijk

**Affiliations:** ^1^ Department of Clinical Genetics Erasmus MC Rotterdam The Netherlands; ^2^ Department of Obstetrics and Fetal Medicine Erasmus MC Rotterdam The Netherlands; ^3^ North Thames Genomic Laboratory Hub Great Ormond Street Hospital London UK; ^4^ Population, Policy and Practice Department UCL Great Ormond Street Institute of Child Health London UK; ^5^ Genetic and Genomic Medicine UCL Great Ormond Street Institute of Child Health London UK; ^6^ Center for Fetal Diagnostics Aarhus University Hospital Aarhus Denmark; ^7^ Department of Clinical Medicine Aarhus University Aarhus Denmark; ^8^ Department of Clinical Genetics Aarhus University Hospital Aarhus Denmark; ^9^ Department of Genetics and Stanford Center for Biomedical Ethics Stanford University School of Medicine Stanford California USA

**Keywords:** chromosomal microarray, health planning guidelines, health policy, practice guidelines, prenatal diagnosis, uncertainty, whole exome sequencing

## Abstract

Exome sequencing (ES) enhanced the diagnostic yield of genetic testing, but has also increased the possibility of uncertain findings. Prenatal ES is increasingly being offered after a fetal abnormality is detected through ultrasound. It is important to know how to handle uncertainty in this particularly stressful period. This systematic review aimed to provide a comprehensive overview of guidelines available for addressing uncertainty related to prenatal chromosomal microarray (CMA) and ES. Ten uncertainty types associated with prenatal ES and CMA were identified and defined by an international multidisciplinary team. Medline (all) and Embase were systematically searched. Laboratory scientists, clinical geneticists, psychologists, and a fetal medicine specialist screened the papers and performed the data extraction. Nineteen papers were included. Recommendations generally emphasized the importance of trio analysis, clinical information, data sharing, validation and re‐analysis, protocols, multidisciplinary teams, genetic counselling, whether to limit the possible scope of results, and when to report particular findings. This systematic review helps provide a vocabulary for uncertainties, and a compass to navigate uncertainties. Prenatal CMA and ES guidelines provide a strong starting point for determining how to handle uncertainty. Gaps in guidelines and recommendations were identified and discussed to provide direction for future research and policy making.

## Graphical abstract

Guidelines for prenatal chromosomal microarray (CMA) and Exome Sequencing (ES) were identified through a systematic review and summarized within a framework of ten defined uncertainty types associated with this prenatal genetic testing. This paper aimed to provide a vocabulary for prenatal genetic uncertainty, as well as a compass to navigate these uncertainties.

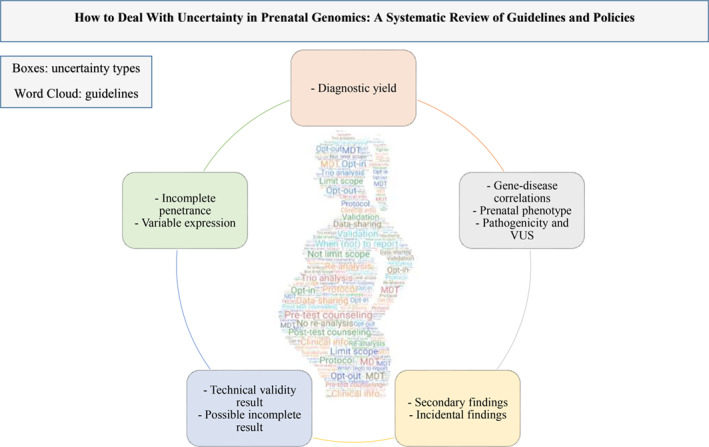

## 1 INTRODUCTION

Technological innovation in prenatal diagnostics ‐ from karyotyping to chromosomal microarray (CMA) and, more recently, from targeted DNA analysis to exome sequencing (ES) ‐ substantially improves diagnosis of previously undetectable genetic anomalies.^1^, ^2^ Currently, some countries are introducing ES in prenatal genetics in cases of fetal malformations,^3^, ^4^ generating large amounts of information on the genome of the unborn child compared to karyotyping, CMA or targeted genetic testing panels. This raises the concern for an increased chance of an uncertain finding, such as genes or variants of uncertain significance (GUS/VUS).^5^ Filters based on the presenting phenotype and for genes with valid phenotypic associations minimize this uncertainty, but may decrease the diagnostic yield. Alternatively, a more open analysis increases the chance of a diagnosis, but also increases the chance of finding an uncertain result.^6^ This can be a challenge for all stakeholders involved. Laboratory specialists are challenged to interpret results. Clinicians are challenged to return results that have an element of uncertainty in a way that is understandable to parents experiencing an extremely stressful diagnostic process. Parents are challenged to apply meaning to this information, and decide about the course of their pregnancy based on results that may not provide the certainty that they had hoped for.^7^, ^8^

Current implementation practices have therefore focused on developing strategies to deal with these uncertainties, both in laboratory and clinical settings.^9^, ^10^ One such strategy is the development of recommendations and guidelines. This systematic review provides an overview of guidelines and recommendations for practice that are available to support professionals dealing with uncertainty in routine clinical prenatal diagnostics.

## 2 MATERIALS AND METHODS

### 2.1 Proposed definitions of uncertainty

The authors conducted multiple discussions on the possible types and definitions of uncertainties that may be encountered during the process of providing ES diagnostics in clinical settings. Ten types of uncertainty associated with prenatal ES, from laboratory and clinical perspectives, are proposed (see Table 1 and S2). In this review we used these definitions to classify recommendations addressing uncertainty and developed a framework for analysing the papers included in this systematic review.

**TABLE 1:** Summary of the types of uncertainties associated with prenatal WES, and their definitions

Type of uncertainty	Subtype	Definition
**1) Uncertainty related to clinical effectiveness**	Diagnostic yield	Likelihood to provide a diagnosis.
**2) Uncertainties related to incomplete knowledge**	Gene‐disease correlations	Phenotype associated with a variant is unknown (prenatally and postnatally), including its variability in expression and the natural history.
	How a genetic anomaly presents prenatally	New phenotypes associated with genes that have limited natural history information in the prenatal period. Or postnatal phenotype associated with pathogenic variant (e.g. mental disability) is not or only partially recognized prenatally.
	Pathogenicity and variants of unknown significance (VUS)	Insufficient evidence to classify variants as (likely) benign or (likely) pathogenic.
**3) Uncertainties unrelated to the primary clinical question**	Secondary findings	Pathogenic variant(s) not related to indication of testing, but intentionally searched for as an additional analysis next to the standard test.
	Incidental findings	Pathogenic variant(s) not related to indication of testing and are identified inadvertently (unexpected result).
**4) Uncertainties related to the technology**	Technical validity of a result	False positives, false negatives, insufficient depth of read.
	Possible incomplete result	For example, One autosomal recessive variant compatible with the fetal phenotype, but no second variant is identified.
**5) Uncertainties related to the condition**	Incomplete penetrance	Chance that a pathogenic variant presents with symptoms. Not everyone with the same genetic predisposition will be affected (reduced or incomplete penetrance).
	Variable expression variants	A pathogenic variant with 100% penetrance where patients with the same variant can show different symptoms (variable expression).

### 2.2 Systematic review of guidelines and recommendations

We conducted a systematic review following PRISMA criteria^11^ to identify guidelines and recommendations addressing uncertainties associated with prenatal diagnostic (genome wide) testing. As prenatal ES is a newly introduced technology we anticipated that there may not be many guidelines available yet and we therefore also included guidelines for prenatal CMA, which might also be relevant to prenatal ES.

### 2.3 Search criteria and study selection

The search was conducted with professional assistance of the Erasmus Medical Centres’ library biomedical information experts across two electronic databases (Embase and Medline Ovid) and Google Scholar on September 29, 2020 (see S1 for the complete search strategy). Additionally, we manually searched reference lists of relevant papers and websites and journals of the following professional societies: ACMG, ACOG, CCMG, ESHG, ISPD, ISUOG, SMFM, SOGC, PQF, NSGC, and BSGM. Duplicates were removed.^12^

Seven assessors were divided into two multi‐disciplinary teams (MDTs), who each independently screened half of the titles and abstracts of the identified papers. Each team consisted of a laboratory specialist (MIS, HTB), clinical geneticist (KEMD, LCPG), and a researcher from psychology (SRR, JEK), all experienced in clinical prenatal genetics (CMA and/or ES). One team also included a gynaecologist (ATJIG). For full‐text eligibility assessment, each team independently assessed half of the selection of papers. In order to identify guidelines on how to deal with uncertain findings within prenatal diagnostic testing we used the following inclusion criteria (PICOS)^13^, ^14^:

- Population addressed: Healthcare professionals (clinicians and laboratory scientists) in the field of prenatal genetics.
- Intervention: Prenatal diagnosis by genome wide techniques such as ES, Genome Sequencing, or CMA.
- Outcome: Guidelines, points to consider, policies, position statements, recommendations
- Study design: Practice guidelines documents, policy or position statements, recommendation papers, points to consider documents, committee opinion papers.

**FIGURE 1:**
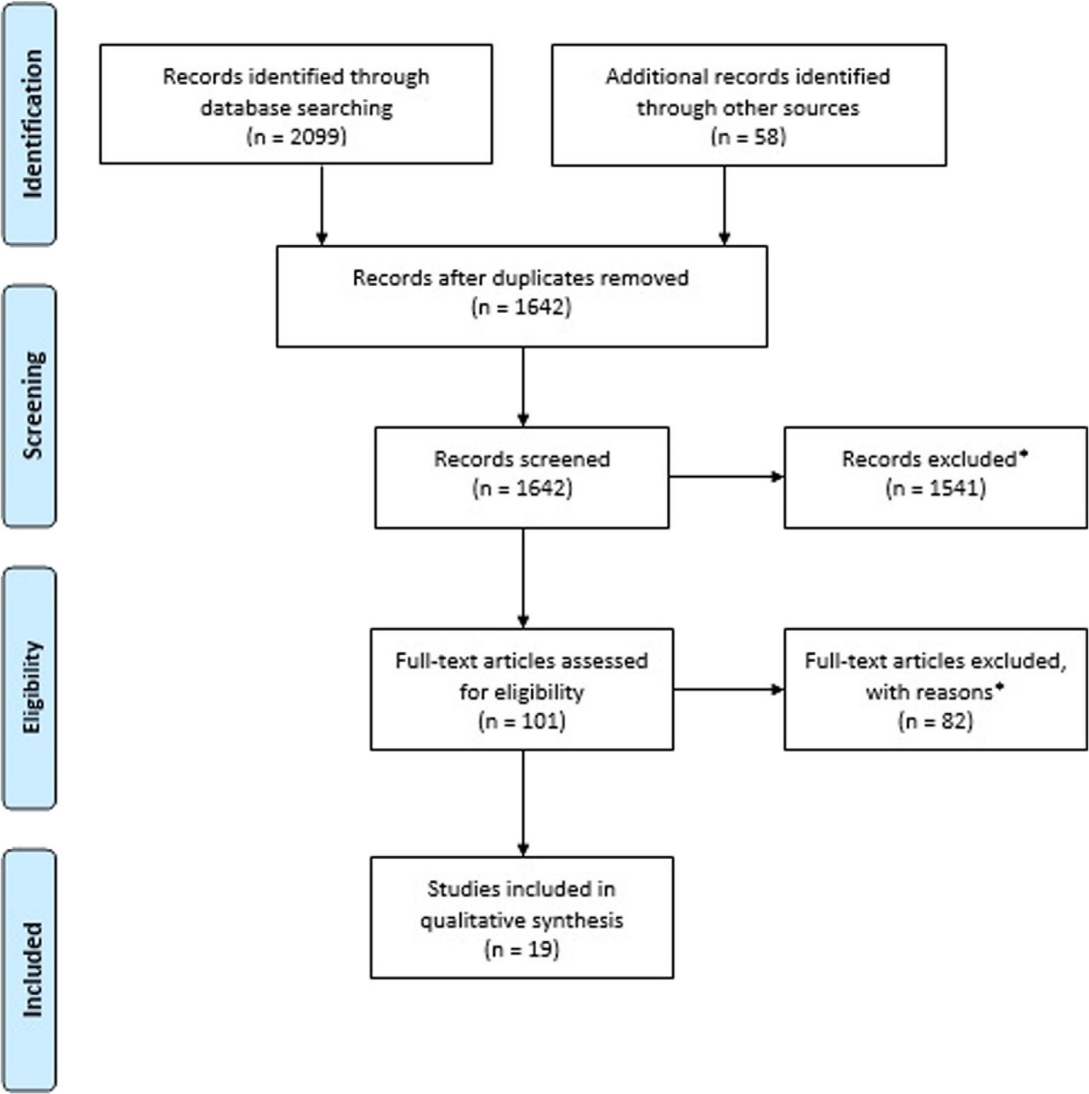
PRISMA flow diagram

We excluded non‐English, retired, and exclusively postnatal papers, as well as papers exclusively on specific syndromes, prenatal screening, predictive testing, preimplantation diagnosis, or genetic testing procedures (also see Figure 1). Disagreements were discussed until consensus was reached on final inclusion.

### 2.4 Data analysis

To assess the quality of identified publications, a modified version of the Appraisal of Guidelines for Research and Evaluation checklist was used.^15^ Each of the seven assessors scored papers independently. The quality scores were not used as a criterion for inclusion. Qualitative data were then analysed using a framework analysis approach.^16^ Each team integrated guidelines according to uncertainty type (Table 1). All seven assessors subsequently convened to discuss the analysis and come to agreement on the coding. Disagreements were resolved by consensus.

## 3 RESULTS

Nineteen papers were eligible for quality assessment and data extraction (see Figure 1). Table 2 summarizes paper characteristics. Ten papers covered ES,^3^, ^4^, ^10^, ^17^, ^18^, ^19^, ^20^, ^21^, ^22^, ^23^ seven papers covered CMA,^24^, ^25^, ^26^, ^27^, ^28^, ^29^, ^30^ and two papers covered both.^31^, ^32^ All seven CMA papers, and two ES papers^4^, ^23^ exclusively described the prenatal setting. The remaining papers incorporated both pre‐ and postnatal settings.^3^, ^10^, ^17^, ^18^, ^19^, ^20^, ^21^, ^22^, ^31^, ^32^ In the following section, recommendations are described by uncertainty type (and summarized in Table 3).

**TABLE 2:** Characteristics of the selected papers

Paper	Y.o.p	Country	Published by	Published in	Type of paper	Technique
ACMG	2012	USA	ACMG	Genetics in Medicine	Policy statement	ES
Armour et al.	2018	Canada	CCMG, SOGC	Journal of Medical Genetics	Policy statement	CMA
Claustres et al.	2014	Multiple countries^a^	ESHG	European Journal of Human Genetics	Policy statement	CMA/ES
Dugoff et al.	2016	USA	SMFM	American Journal of Obstetrics and Gynecology	Consult series	CMA
Hegde et al.	2015	USA	AMP	The Journal of Molecular Diagnostics	Special article	ES
ISPD, SMFM, PQF	2018	International	ISPD, SMFM, PQF	Prenatal Diagnosis	Position statement	ES
MacArthur et al.	2014	USA	US National Human Genome Research Institute	Nature	Guidelines	ES
Matthijs et al.	2016	Belgium, NL, Germany	ESHG	European Journal of Human Genetics	Policy statement	ES
Richards et al.	2015	USA	ACMG, AMP	Genetics in Medicine	Standards and guidelines	ES
Silva et al.	2019	Portugal, NL, UK	ESHG	European Journal of Human Genetics	Policy statement	CMA/ES
Suela et al.	2017	Spain	AEDP, AEGH, SEGCD	Medicina Clinica	Consensus statement	CMA
Vanakker et al.	2014	Belgium	All Belgium genetic centers	European Journal of Medical Genetics	Consensus statement	CMA
Van El et al.	2013	Multiple countries^b^	ESHG	European Journal of Medical Genetics	Policy statement	ES
Vears et al.	2018	Multiple countries^c^	ESHG	European Journal of Medical Genetics	Points to consider	ES
Wallis et al.	2013	UK, NL	ACGS (BSGM), VKGL	Association for Clinical Genetic Science; Dutch Society of Clinical Genetic Laboratory Specialists	Practice guidelines	ES
Monaghan et al.	2020	USA	ACMG	Genetics in Medicine	Points to consider	ES
Gardiner et al.	2015	UK	RCP, BSGM, RCOG, BMFMS (JCGM)	Royal College of Pathologists	Recommendations	CMA
Vetro et al.	2012	Multiple countries^d^	ESHG	Human Mutation	Special article	CMA
Skirton et al.	2014	UK	ESHG	European Journal of Human Genetics	Policy statement	CMA

^a^ France, Czech Republic, Belgium, Switzerland, Netherlands (NL), Germany, Slovenia, Italy, UK, Ireland.

^b^ NL, Belgium, UK, Switzerland, France, Canada, Denmark.

^c^ Belgium, Canada, UK, Slovenia, France, NL.

^d^ Italy, NL, UK, Belgium, Germany, USA.

**TABLE 3:** Summary of recommendations; how to deal with uncertainties

Paper	CMA or ES	Diagn. yield	Gene‐disease correlations	Prenatal presentation genetic anomaly	Pathogenicity (VUS)		Secondary findings		Incidental findings		Technical validity of a result	Possible incomplete result	Penetrance	Expression
**Vanakker et al., 2014**	*CMA*	Limit‐S Pre‐T	x	x	MDT Trio	(Not‐)R*	x		MDT	(Not‐)R*	x	x	Clin‐Info Post‐T	Clin‐Info (Not‐)R*
**Armour et al., 2018**	*CMA*	Limit‐N Pre‐T	x	x	(Not‐)R*		x		Pre‐T Post‐T	(Not‐)R*	x	x	Trio (Not‐)R*	Trio (Not‐)R*
**Dugoff et al., 2016**	*CMA*	x	x	x	Trio	Pre‐T Post‐T (Not‐)R*	x		Pre‐T		x	x	x	x
**Suela et al., 2017**	*CMA*	Pre‐T	x	x	Trio	Pre‐T	x		Pre‐T		Val	x	Clin‐Info Pre‐T	Clin‐Info Pre‐T
**Vetro et al., 2012**	*CMA*	Limit‐N MDT Trio Pre‐T Post‐T	x	Data‐S Clin‐Info	Data‐S MDT Clin‐Info	Trio Pre‐T (Not‐)R*	MDT Clin‐Info	Pre‐T	Limit‐S MDT Protocol	Pre‐T (Not‐)R* Opt‐I	x	x	x	x
**Skirton et al., 2014**	*CMA*	Limit‐S Pre‐T Post‐T	x	x	x		x		Limit‐S Pre‐T		x	x	Post‐T	Post‐T
**Gardiner et al., 2015**	*CMA*	x	x	x	Data‐S	MDT Trio	Post‐T		MDT	Post‐T (Not‐)R*	x	x	(Not‐)R*	x
**Claustres et al., 2014**	*CMA/* *ES*	MDT Protocol Post‐T	x	x	Data‐S Clin‐Info Trio	(Not‐)R*	x		Protocol		Clin‐Info	x	x	x
**Silva et al., 2019**	*CMA*	x	x	x	x		x		Pre‐T		Val	x	x	x
	*ES*	Pre‐T Post‐T	x	x	Pre‐T Post‐T		x		Pre‐T		Val Post‐T	x	x	x
**ISPD, SMFM, PQF, 2018**	*ES*	Pre‐T	x	Data‐S Clin‐Info	MDT Clin‐Info Trio	Pre‐T Post‐T (Not‐)R*	Pre‐T		Pre‐T		x	x	x	x
**Monaghan et al., 2020**	*ES*	Val MDT Protocol Trio Pre‐T Post‐T	x	Clin‐Info	Re‐Analyze MDT Clin‐Info Protocol Trio	Pre‐T Post‐T (Not‐)R* Opt‐O	Protocol	Pre‐T Opt‐O	Limit‐S Protocol	Pre‐T (Not‐)R* Opt‐O	x	x	x	x
**ACMG, 2012**	*ES*	Pre‐T	(Not‐)R*	x	Data‐S	Pre‐T	x		MDT Protocol	Pre‐T (Not‐)R* Opt‐O	x	x	x	x
**Hegde et al., 2015**	*ES*	Pre‐T Post‐T	x	x	Data‐S	Pre‐T	Val Limit‐S Limit‐N Protocol	Pre‐T (Not‐)R* Opt‐I Opt‐O	Protocol	Pre‐T (Not‐)R* Opt‐I Opt‐O	Post‐T	x	x	x
**MacArthur et al., 2014**	*ES*	x	Clin‐Info	x	Data‐S	Clin‐Info Trio	x		x		Val Data‐S	x	Data‐S Clin‐Info	Data‐S Clin‐Info
**Matthijs et al., 2016**	*ES*	x	Limit‐S	x	Re‐Analyze(‐n)	Data‐S Protocol	Limit‐S		Limit‐S Protocol	Pre‐T	Val	x	x	x
**Richards et al., 2015**	*ES*	x	Clin‐Info	Post‐T Clin‐Info	Clin‐Info Protocol		x		x		x	x	Clin‐Info	Clin‐Info
**Van El et al., 2013**	*ES*	x	x	x	Pre‐T		Limit‐S		Protocol	(Not‐)R*	x	x	x	x
**Vears et al., 2018**	*ES*	x	Data‐S Clin‐Info	x	Re‐Analyze(‐n)	Data‐S Clin‐Info	Limit‐S	Pre‐T	Pre‐T	(Not‐)R* Opt‐O	x	x	Trio	Trio
**Wallis et al., 2013**	*ES*	x	MDT Clin‐Info	x	Val Data‐S	MDT Clin‐Info Protocol	x		x		x	x	x	x

Recommendations pertaining to lab technicians	
Val = Validation with other technique is recommended or determination for various thresholds	Re‐Analyze(‐n) = Re‐analysis when new information is available (should not be done routinely or only under described circumstances).
Data‐S = Data storage or data sharing (and re‐evaluation) is recommended.	
Recommendations pertaining to lab technicians and clinicians	
Limit‐S = The scope should be limited (e.g. by filtering, lowering resolution).	Limit‐N = The scope should not be limited.
MDT = Discuss within multidisciplinary team how to handle the uncertainty.	Clin‐Info = Supply clinical information/use multiple lines of evidence.
Protocol = A protocol, policy or set of agreed standards should be in place.	Trio = Also sequence the probands’ parents.
Recommendations pertaining to patients and clinicians	
Pre‐T = Obtain informed consent or information on diagnostic possibilities should be stated in informed consent document, patients leaflet or given during pre‐test counselling.	Post‐T = Issue to discuss during post‐test counselling after reporting. Information about limitations of the test or a disclaimer should be included in the report (to the clinician).
(Not‐)R* = Advice to (not) report, with specific exceptions.	Opt‐I/Opt‐O = Advice to allow opt in/out.

### 3.1 Uncertainty related to clinical effectiveness

#### 3.1.1 Diagnostic yield

The analysis resolution defines the diagnostic yield of CMA, which should be incorporated in the laboratory report and in the informed consent.^26^ Resolution recommendations varied between 200 kb resolution,^24^, ^29^ and 400 kb to minimize VUS and maximize yield.^27^ Skirton et al (2014) recommended targeted testing when employing CMA to lower the chance of finding VUS.^30^ Trio analysis is recommended to improve diagnostic yield^23^ and obtain results faster, which is especially important in the prenatal context.^29^ Segregation analysis on parental samples or samples from close relatives should be considered if trio analysis is not possible.^23^ Parental results should be reported separately from the fetal results.^32^

Lab and clinicians should work together in deciding on methods and available testing options.^23^, ^29^ Protocols should prescribe turnaround times, what should minimally be interpreted in the laboratory report, and what should be discussed in a multi‐disciplinary team (MDT),^32^ and whether re‐analysis with an updated report is provided.^23^

Pre‐test genetic counselling and the informed consent process should discuss realistic expectations about the likelihood of a diagnosis, the possibility of not obtaining a result before birth, as well as the scope, and resolution of the test.^3^, ^4^, ^17^, ^24^, ^26^, ^27^, ^29^, ^30^, ^31^, ^32^ Post‐test counselling should include a discussion of limitations (e.g. poor detection of certain variants/coverage) of the test,^17^, ^29^, ^30^, ^31^ and options for pregnancy management should be explained.^30^ Parents should also be informed in pre‐ and post‐test counseling that not finding a causative variant for the primary indication is a possibility, and does not mean a genetic cause was ruled out.^17^, ^23^ If no genetic cause is found for the fetal abnormalities, post‐test counselling should explain the residual risk.^23^, ^29^

### 3.2 Uncertainties related to incomplete knowledge

#### 3.2.1 Gene‐disease correlations

Matthijs et al (2016) stated that in a diagnostic ES setting, analysis should only include genes with established phenotype‐genotype correlations.^19^ Overall evidence for variant or gene implication should be assessed and integrated, including primarily statistical support (genetic analyses) and, if possible, informatic (e.g. conservation and predicted effect on function) and experimental evidence (e.g. functional studies).^18^ To confidently implicate a new gene in disease, these genes should be replicated in independent families or population cohorts. Null models (for e.g. de novo variants) should be used to compare against when detecting pathogenic variants, while also considering potential confounders (e.g. sample or gene size).^18^

Detailed information on the phenotype is necessary to interpret the genotype, making clinical information important for the laboratory analysis as well as in the decision to report (to the clinician),^20^ and should be available to the MDT before sequencing.^10^, ^22^ Providing all clinical information is not always feasible when time is limited.^10^ If there is compelling information implicating a variant in a proband's phenotype it could be included in the test results.^3^ Sharing phenotypic data paired with variants in databases further improves gene and variant interpretation.^10^

#### 3.2.2 How a genetic anomaly presents prenatally

Prenatal genotype‐phenotype correlations are often identified as uncertain because of limited information of the prenatal phenotype.^4^, ^29^ Many known microdeletions/microduplication syndromes were identified postnatally with limited data on the prenatal presentation of many of the syndromes,^31^ or there was biased ascertainment,^29^ typically of the more severely affected fetuses. Clinical information is important and should be submitted in standardized format, with imaging data as support for the fetal phenotypic findings.^4^, ^20^ Monaghan et al (2020) provided an extensive description of what clinical information should be provided, including: ''detailed fetal imaging reports […], prior fetal prenatal test results and/or clinical laboratory report, parental past medical history, ethnicity, reproductive history, and family history, including parental consanguinity.''^23^ Collecting (phenotypic) data allows for improved correlations between genetic data and a potential disease, although this is complicated when crucial clinical characterization of the fetus is not possible or non‐specific.^29^ Laboratories should set up systems where this clinical information can be submitted.^4^ If the genetic data are the only other information available, post‐test counseling was considered especially important.^20^

#### 3.2.3 Pathogenicity and variants of unknown significance

##### Careful classification of pathogenicity of variants

Careful classification of variants is crucial for correct reporting. Sequence variants should be reported using the classification system as proposed by Richards et al (2015), distinguishing five classes (pathogenic variants, likely pathogenic variants, variants of uncertain significance or unclassified variants, likely benign variants and benign variants).^20^ Classification should be based on agreed standards and informed by multiple lines of evidence, including clinical information and empirical data.^10^, ^18^, ^20^, ^22^ Variants in candidate genes should not be classified higher than VUS.^23^

Gardiner et al (2015) stated that it should be decided in MDT whether trio analysis is appropriate when employing CMA.^28^ Others however, generally advised trio analysis to aid interpretation and decision‐making concerning reporting,^4^, ^25^, ^26^, ^27^, ^29^ by improving variant classification,^18^ discovering de novo variants or compound heterozygosity efficiently,^23^ or formally confirming a diagnosis.^32^

Clinical information was considered imperative to aid the interpretation of variants,^20^, ^22^ as were the prenatal phenotype and MDT discussions about the phenotypic information.^4^, ^22^, ^23^ With regards to the laboratory report, several elements should be considered; (1) determination of cis or trans of variants in case of (potentially) multiple pathogenic variants, (2) information obtained from, for example, literature, prediction programs, or databases, which should clearly support assigned pathogenicity, (3) integrated individual results in case of multiple analyses on one sample, (4) references that were used in interpreting results where appropriate (e.g. rare or unclassified variants), (5) whether the clinician should supply missing information that may help in the (accuracy of) interpretation, (6) significance of the finding(s).^32^

It was recommended that clinical laboratories submit (de‐identified) variant data to public databases, including data on potential pathogenicity, VUS, relevant clinical information, and frequency data.^3^, ^10^, ^17^, ^19^, ^22^ Information in these databases should be updated and re‐evaluated continuously.^17^, ^18^ Such databases and sharing within these databases will, over time, reduce the amount of VUS as more data are pooled together on unknown variants improving identification and classification of variants.^10^, ^19^ This data sharing should be an integral part of reporting and thus supported by an international committee.^32^

Old data should not be routinely re‐analysed.^10^, ^19^ However, it was recommended to issue a new report to the clinician when categorization of a variant changes significantly, for example from (likely) pathogenic to (likely) benign.^10^ This possibility should be communicated with parents during post‐test counselling.^23^ Other re‐analyses can be requested by the patient through their clinician, especially in case of a future pregnancy, after considerable time (e.g. more than 12 months),^10^, ^23^ if the ES report did not comprise a complete phenotype, or the phenotype has expanded postnatally. Furthermore, new gene‐disease correlations might have been established and the fetal phenotype may now turn out to be correlated with the genotype.^23^

##### Considerations toward reporting

Some CMA guidelines discouraged reporting VUS.^24^, ^27^ According to Richards et al (2015) VUS in sequence variants should not be used in clinical decision making.^20^ To minimize the need for analysing and reporting VUS, Armour et al (2018) suggested that VUS should not be reported except for deletions of >500 kb and duplications of >1 Mb if there is emerging evidence for pathogenicity.^24^ Vetro et al (2012) recommended not reporting VUS not associated with an ultrasound abnormality, and to be cautious in deciding to report copy number variants (CNV's) that are unlikely or unknown to have caused the structural abnormality.^29^ However, Claustres et al (2014) recommended to include VUS in the report in case they may become clinically significant in the future.^32^ There is debate on whether to report VUS that may contribute to the abnormal fetal phenotype.^4^, ^23^ A publication on prenatal ES stated that reporting of VUS that fit the prenatal phenotype should be considered.^23^ Finally, Suela et al (2017) suggested that patients should be offered a choice in which results to receive,^26^ while Dugoff et al (2016) suggested reporting VUS, but only after extensive pre‐ and post‐test counselling.^25^

Guidelines were not always clear on how to handle VUS, but often depended on local practice.^19^, ^20^, ^22^, ^23^ What practice or protocol is in place should be clear to laboratory scientists and clinicians.^19^, ^23^ It was considered helpful to form a network of laboratories that can share data and to consult with.^29^ A multi‐disciplinary committee has to be available to discuss difficult cases,^4^, ^27^, ^28^ and it was recommended to keep MDT in place, even if the need decreases as experience with new techniques (e.g. through data sharing) evolve.^28^ Before offering testing, the laboratory and the clinicians should confer and agree on what to report, both to the relevant clinicians and the parent(s).^29^

Patients should be informed about the possibility of finding/reporting VUS and other potential outcomes beforehand.^3^, ^4^, ^21^, ^23^, ^29^, ^31^ Pre‐ and post‐test counselling were again considered important in guiding patients through uncertainty.^4^, ^25^, ^31^ During pre‐test counselling, a genetic healthcare professional should obtain clinical information and consent.^29^ The types of variants that will be reported should be included in the consent document, including how this is different for the reporting policy of incidental findings (IF's) and secondary findings (SF's).^17^ Parents should be able to opt‐out of receiving variants in non‐disease genes.^23^ If VUS were reported, it should be made clear to the parent(s) that other laboratories may have different policies for reporting VUS prenatally, and VUS may not be reported after future (targeted) testing.^23^

### 3.3 Uncertainties unrelated to the primary clinical question

#### 3.3.1 Secondary findings

The ACMG SF list initially excluded the prenatal setting,^33^ however a more recent ACMG recommendation specifically on prenatal ES prescribed that there should be clear policies in place to elucidate (1) whether only SF's in the fetus are reported or also SF's in the parents, and (2) whether analyzing, filtering, and variant calling of SF's in parents should be limited to those found in the fetus.^23^ Several European guidelines were not supportive of actively searching for SF's, and recommended to target the analysis to the genes related to the primary indication.^10^, ^19^, ^21^ A laboratory's protocol should be based on whether they are able to provide information with enough accuracy, and clinicians should address which SF's are routinely analyzed and reported as part of the informed consent procedure.^17^ Clinical information is important to support variant interpretation, as an SF may not be unexpected (i.e. based on family pedigree).^29^

Generally, laboratories should only report known or (likely) pathogenic SF's.^17^ Limiting the scope of testing may be possible for complex findings (e.g. pseudogenes), but this may be undesirable for SF's on the ACMG list.^33^, ^34^ In those cases only variants on the active copy of the gene should be reported, and validated with additional testing (i.e. Sanger sequencing) if needed.^17^

Vears et al (2018) suggest that analysis for SF's (thus searched for), should be performed separately and with informed consent from the patient.^10^ In general, patients should be informed about (1) whether other genes are analyzed that are not related to the phenotype (e.g. ACMG SF gene list), (2) whether patients can opt‐in or out of receiving SF's, (3) how reporting of SF's differs from reporting variants that are related to the primary indication, and (4) how sequenced individuals receive SF's in case of trio analysis.^17^ In trio analysis, every sequenced person (other than the proband) should give separate consent.^17^ The chance of finding SF's and whether SF's are included or excluded should be discussed during pre‐test counselling and during the informed consent procedure.^4^, ^23^, ^29^ Offering an opt‐out of receiving SF's (from the ACMG list) is recommended.^23^ Vetro et al (2012) recommended the MDT to decide about an opt‐in to receive (likely) pathogenic SF's. This choice should be clearly communicated to the laboratory.^29^ If treatable pathogenic SF's are reported, it should be clearly explained that they are unrelated to the primary indication.^28^

#### 3.3.2 Incidental findings

The chance of IF's can be reduced by focusing on the gene panel under investigation or by targeted testing.^19^, ^30^ A targeted or (whole‐)exome approach should be in line with the referral reason and the informed consent.^31^ Only variants found in the fetus should be analyzed in parental arrays to avoid detecting IF's in the parents.^23^, ^29^ There should also be a clear protocol or policy in place on whether IF's are reported,^21^, ^23^, ^29^, ^32^ and/or an opt‐in or out is offered.^3^, ^17^, ^19^ Local policies should be in place for reporting IF's and non‐paternity to the clinician,^23^ because if parents decide to continue the pregnancy, then prenatal diagnosis is a form of early presymptomatic testing. Policy should take into account the future child's autonomy and right to an open future, as well as parental interests, rights, and needs.^21^

IF's of uncertain significance, or without health implications, should not be reported.^10^, ^24^, ^28^ Prenatal ES guidelines stated to (1) not report variants without a known fetal or childhood phenotype, (2) not report when heterozygous for autosomal recessive disorders or X‐linked disorders, and (3) report highly penetrant pathogenic IF's that may cause moderate or severe early‐onset disorders.^23^ Armour et al (2018) suggested that if parents indicated they want to know all relevant results, laboratories should report pathogenic IF's.^24^ Other papers recommended to only report known and (likely) pathogenic IF's,^3^, ^17^, ^23^ or (likely) pathogenic, actionable IF's.^21^, ^28^ Early onset disorders are usually reported, but late onset disorders are not.^27^ An opt‐in for treatable late‐onset disorders, but not for non‐treatable late‐onset IF's may be offered.^29^ Deletions in genes found through CMA that are associated with recessive disorders not fitting the fetal phenotype should only be reported if the carrier frequency is higher than 1:50.^27^ Vears et al (2018) proposed to report heterozygosity of recessive disorders, but to honor an adults' informed consent and/or choice to opt‐out, even if the IF may be relevant to their health.^10^

Pre‐test counseling should address the possibility of IF's and which IF's are (not) reported,^3^, ^4^, ^17^, ^19^, ^23^, ^24^, ^25^, ^26^, ^29^, ^31^ Pre‐test counseling should also discuss the possibility to detect non‐paternity and how this influences the interpretation of the genetic results.^29^ Matthijs et al (2016) recommended supplementing this information with written leaflets or online information.^19^ Patients should be able to opt‐in or out of receiving IF's and their choice should be clearly indicated^3^, ^10^, ^17^, ^23^ to prevent disclosure of unwanted results.^29^ Extensive post‐test counseling should be offered when reporting pathogenic IF's.^24^ Difficult IF cases need to be discussed by a MDT on a case‐by‐case basis.^3^, ^4^, ^27^, ^28^, ^29^ The psychological impact and potential insurance risks of receiving such findings should be taken in consideration.^17^ If treatable pathogenic IF's are reported, it should be made clear that these findings are not associated with the indication of testing.^28^

### 3.4 Uncertainties related to the technology

#### 3.4.1 Technical validity of a result

In case of a finding based on insufficient read‐depth or ''noisy'' CNV's validation by a different technique was recommended.^26^, ^31^ To reduce the impact of unwanted false positives meticulous evaluation and subsequent re‐evaluation of candidate variants in databases was deemed important.^18^ If possible, estimated diagnostic specificity may be provided in the report to the clinician to indicate the risk of a false positive.^32^ The limitations of enrichment methods and sequencing platforms should determine whether additional testing is required and, if so, what type of additional testing is required.^19^ Disclaimers and limitations concerning coverage should be clearly described in the laboratory report.^17^, ^31^

#### 3.4.2 Possible incomplete result

This situation was not specifically discussed by any of the guidelines that were reviewed.

### 3.5 Uncertainties related to the condition

#### 3.5.1 Penetrance and expression

Penetrance and expression were rarely discussed in ES guidelines. MacArthur et al. (2014) described the importance of assessing disease‐associated variants in large, well‐phenotyped population cohorts in order to obtain accurate data on penetrance and expression estimates.^18^ Also in case of incomplete penetrance and variable expression, clinical information can aid variant interpretation.^20^ Due to the indirect and limited establishment of the phenotype of the fetus, it is difficult to diagnose disorders with incomplete penetrance in the absence of evidence or family history.^26^, ^27^

For both ES and CMA, trio analysis was recommended to aid clinical interpretation.^10^, ^24^ Incomplete penetrance requires caution as a parent carrying a certain variant may not be affected, while the variant may be(come) penetrant in the fetus.^29^ Susceptibility CNVs, which are associated with both variable expression and variable penetrance, are especially challenging and recommendations strongly differ; if high penetrance neuro‐susceptibility loci are found with CMA that may be associated with a severe phenotype, Gardiner et al recommended reporting these.^28^ Others suggested reporting only some, depending on the penetrance and fetal phenotype,^24^, ^27^ while Suela et al (2017) suggested discussing these findings during pre‐test counselling and allowing parents to decide on the report.^26^ The implications of incomplete penetrance and variable expression should be explained to parents.^26^, ^27^, ^30^

## 4 DISCUSSION

This systematic review provides an overview of existing guidelines for dealing with uncertainty in prenatal CMA and ES. Generally, recommendations emphasize the importance of local policy and protocols, providing clinical information, and using trio analysis to aid in interpretation, the use of databases and data sharing, validation of results, discussion of findings in MDTs, re‐analysis of data, pre‐ and post‐test counselling, as well as providing guidelines on when to report findings, and whether to limit the possible scope of results. There were areas that require further attention and some gaps in the available guidelines and recommendations were identified.

### 4.1 Local policy and protocols

Local decision‐making was highlighted as an important factor in handling uncertainty. The decision to report problematic variants (VUS, IF's, SF's, etc.) for example, often depends on MDT and/or policies that are put in place either by a country or laboratory. Guidelines were therefore often more general and non‐specific (e.g. report treatable (late‐onset) IF's, but not non‐treatable IF's). Rarely were recommendations given on what a policy should specifically look like or include. This acknowledges that local policies exist on how to handle uncertainties and recognizes that there are differences between laboratories and healthcare contexts. With global institutions using different protocols and policies that are often only known internally, it is difficult to achieve an overview of all the local policies that currently exist. Developing more universal guidelines incorporating policies that can apply within each institution is therefore challenging and may even be unnecessary.

### 4.2 Providing clinical information

The importance of clinical information has been mentioned in the context of most uncertainty types (see Table 3). However, it should be kept in mind that it is not feasible to provide all the clinical information on the fetus due to the situation depending on imaging techniques, the fact that development and function of organs is incomplete, for instance of the brain, and time constraints. New features may become evident after birth, which may lead to re‐interpretation of prenatal results.^35^

### 4.3 Use of databases and data sharing (including validation and re‐analysis)

Clinical and empirical evidence is important to classify causative results. Empirical data should be fed into and retrieved from shared databases, which serve as a platform for knowledge building and can help interpret variants that are of uncertain or unknown significance.^10^, ^19^ However, some issues on use of databases and data sharing were not explicitly discussed in the guidelines: (1) studies may be biased, e.g. data of a particular population, (2) curation of the databases is a very important factor and data should be validated and updated continuously at a rapid pace. This requires extensive effort and depending on available resources may not always be feasible,^36^ (3) data sharing can be done nationally and/or internationally. Sharing data within an extended network (i.e. centralizing) will enable optimal knowledge building, but requires calibration of different systems. Yet, there are already some publicly available international databases that are used to share anonymized pathogenic variants and data originating from healthy individuals.^29^ (4) None of the guidelines mention follow‐up either after birth or upon termination of pregnancy. Follow‐up information could be useful in providing knowledge about the development of the prenatal phenotype of a certain variant. There is still much to learn, and especially for the rarely discussed uncertainty types where incomplete knowledge is an issue (e.g. How a genetic anomaly presents prenatally, and gene‐disease correlations), updating databases regularly with clinical information and follow‐up data can prove instrumental in increasing diagnostic yield.^37^

### 4.4 Multi‐disciplinary team

An MDT approach is the norm when it concerns handling particularly uncertain findings. The MDT serves to aid the lab specialist and referring clinical geneticist to interpret results and determine whether to report uncertain findings (e.g. where pathogenicity is unclear). The MDT should include representatives of all needed disciplines that can bring technical, empirical, and clinical insights together. This is mostly discussed locally, but the MDT can be useful on a larger scale as well. Extending the MDT network to include, for example, other laboratories or centres can help with optimizing data sharing and expands the consultation options.^29^ The latter is also the case when using MDT on an international level, enabling collaboration of an expert group that can then serve as an additional resource.^28^

### 4.5 Pre‐ and post‐test counselling

Generally, recommendations stated that uncertainties should be addressed during pre‐ or post‐test counselling. Also, pre‐ and post‐test counselling should enable parents to make informed decisions about which results they wish to receive. Only two papers offered more specific direction on which points should always be discussed in pre‐ and/or post‐test counselling and in which format(s) (e.g. written and orally).^28^, ^29^, ^30^ Several papers agreed counselling should be provided by a specialized genetic professional.^3^, ^4^, ^23^, ^25^, ^30^, ^32^ Offering psychosocial care during counselling was rarely recommended, while the prenatal setting causes significant psychological distress as parents are challenged to make (irrevocable) decisions about their pregnancy.^38^

### 4.6 Implications of guidelines for patients

Although guidelines were often rigorously developed, input of parents on guidelines was rarely sought out. This may be the case, because these guidelines are aimed at the healthcare professionals. Uncertainty that may have originated in the laboratory for example occurs mostly behind the scenes before the result reaches the parents. However, as guidelines reflect different views of healthcare professionals on how to handle uncertain results (e.g. reflected in the least amount of consensus on VUS, which is associated with the largest amount of uncertainty), they also reflect the views of patients. Especially when the importance of counselling is highlighted by most recommendation papers, it may be worthwhile to include the patient as part of the MDT when developing guidelines.

### 4.7 Strengths and limitations of the systematic review

This systematic review was strengthened by the participation of an MDT of experts in reviewing the guidelines. Another strength is the proposal of 10 distinguishable uncertainty types. International MDT discussions were held until there was consensus on clear and mutually exclusive definitions, which were used to identify guidelines as well as provide vocabulary to internationally discuss prenatal ES uncertainties.

A limitation of the systematic review is the comparison of prenatal CMA and ES guidelines. CMA was included because a lack of guidelines was expected for prenatal ES. However, there are diagnostic differences between CMA and ES. ES has a higher resolution, which has the advantage of widening the diagnostic yield, but at the same time there is a greater chance to encounter VOUS, and IF's. Manual inspection of variants is more feasible with CMA, while ES depends more on variant filtering. Lastly, older CMA guidelines may have classified CNV's and single nucleotide variants differently. These differences should be considered when extrapolating guidelines to prenatal ES. Nonetheless, there is still much to learn for prenatal ES and lessons learned from the use of prenatal CMA should be taken into account. Omitting these guidelines would have significantly limited the overview of what guidance is currently available.

### 4.8 Directions of future research

Recommendations that are in place prenatally form a sound starting point on how to handle uncertainty. However, to inform future guidelines and policies, elaborate discussions could be initiated between various stakeholders, both locally and internationally, on what have been best practices in their experience and which of these would (not) hold in the prenatal ES setting. Secondly, seeing as there are cultural and legislation differences between countries, it may not be realistic to develop universal guidelines. Nonetheless, research into these differences, but also similarities, between countries may provide valuable insight into what guidelines are universally applicable and which parts of the process are in need of localized guidelines and what these should be.

## CONFLICT OF INTEREST

The authors declare no potential conflict of interest.

### PEER REVIEW

The peer review history for this article is available at https://publons.com/publon/10.1111/cge.14010.

## Supporting information


**Appendix** **S1: Supporting information**

## Data Availability

Data sharing not applicable to this article as no datasets were generated or analysed during the current study.
