# Supplementary material for: How to deal with uncertainty in prenatal genomics: A systematic review of guidelines and policies
Source: Clin Genet. 2021 Jun 30;100(6):647–58. doi: 10.1111/cge.14010 (PMC8596644; doi:10.1111/cge.14010)
Supplement: Supplementary file 1 — Appendix S1: Supporting information [file CGE-100-647-s001.docx]

**Supplemental material**

**How to deal with uncertainty in prenatal genomics: A systematic review of guidelines and policies**

Jasmijn E. Klapwijk^1^, Malgorzata I. Srebniak^1^, Attie T.J. I. Go^2^, Lutgarde C.P. Govaerts^1^, Celine Lewis^3,4^, Jennifer Hammond^3,5^, Melissa Hill^3,5^, Stina Lou^6^, Ida Vogel^6,7,8^, Kelly E. Ormond^9^, Karin E. M. Diderich^1^, Hennie T. Brüggenwirth^†1^, Sam R. Riedijk^†1^

^†^*Hennie T. Brüggenwirth and Sam R. Riedijk should be considered joint senior author.*

^1^Department of Clinical Genetics, Erasmus MC, Rotterdam, The Netherlands

^2^Department of Obstetrics and Fetal Medicine, Erasmus MC, Rotterdam, the Netherlands

^3^North Thames Genomic Laboratory Hub, Great Ormond Street Hospital, London, UK

^4^Population, Policy and Practice Department, UCL Great Ormond Street Institute of Child Health, London, UK

^5^Genetic and Genomic Medicine, UCL Great Ormond Street Institute of Child Health, London, UK

^6^Center for Fetal Diagnostics, Aarhus University Hospital, Aarhus, Denmark

^7^Department of Clinical Medicine, Aarhus University, Aarhus, Denmark

^8^Department of Clinical Genetics, Aarhus University Hospital, Aarhus, Denmark

^9^Department of Genetics and Stanford Center for Biomedical Ethics, Stanford University School of Medicine, Stanford, CA, USA

**Supplement 1**

Complete search strategy

**Search terms medical library**

**embase.com**

('sequence analysis'/exp OR 'gene sequence'/de OR 'DNA sequence'/de OR 'microarray analysis'/exp OR 'tissue microarray'/de OR 'DNA microarray'/de OR 'high throughput sequencing'/de OR (sequenc* OR gene-panel* OR microarray OR array OR arrays OR CMA):ab,ti) AND ('practice guideline'/de OR 'consensus development'/de OR 'medical society'/de OR 'policy'/exp OR (guideline* OR ((consensus OR position*) NEAR/6 (national* OR international* OR association* OR Committee* OR opinion* OR network* OR countr* OR process* OR development* OR expert* OR Gynaecologist* OR Obstetrician* OR working-group* OR societ*)) OR recommendation* OR policy OR policies OR statement* OR (guide NEAR/3 clinician*) OR medical-societ* OR national-societ* OR ((acmg OR eshg OR ispd OR ccmg OR acog OR isuog OR smfm OR rcog) NOT (copyright* NEAR/3 (acmg OR eshg OR ispd OR ccmg OR acog OR isuog OR smfm OR rcog)))):ab,ti) AND ('prenatal diagnosis'/exp OR 'prenatal development'/exp OR 'first trimester pregnancy'/de OR 'second trimester pregnancy'/de OR 'third trimester pregnancy'/de OR 'prenatal care'/de OR embryo/exp OR 'fetus'/de OR (prenatal* OR pre-natal* OR foetal OR foetus OR fetal OR fetus OR embryo* OR ((pregnan* OR first OR second OR third OR 1st OR 2nd OR 3rd OR 1-st OR 2-nd OR 3-rd) NEAR/3 trimester*)):ab,ti) NOT ([animals]/lim NOT [humans]/lim) AND [English]/lim

**Medline Ovid**

(exp Sequence Analysis/ OR Microarray Analysis/ OR (sequenc* OR gene-panel* OR microarray OR array OR arrays OR CMA).ab,ti.) AND (guideline/ OR consensus/ OR Societies, Medical/ OR Guidelines as Topic/ OR policy/ OR (guideline* OR ((consensus OR position*) ADJ6 (national* OR international* OR association* OR Committee* OR opinion* OR network* OR countr* OR process* OR development* OR expert* OR Gynaecologist* OR Obstetrician* OR working-group* OR societ*)) OR recommendation* OR policy OR policies OR statement* OR (guide ADJ3 clinician*) OR medical-societ* OR national-societ* OR ((acmg OR eshg OR ispd OR ccmg OR acog OR isuog OR smfm OR rcog) NOT (copyright* ADJ3 (acmg OR eshg OR ispd OR ccmg OR acog OR isuog OR smfm OR rcog)))).ab,ti.) AND (exp Prenatal Diagnosis/ OR exp Embryonic Structures/ OR exp Pregnancy Trimesters/ OR Prenatal Care/ OR exp Embryology/ OR exp fetus/ OR (prenatal* OR pre-natal* OR foetal OR foetus OR fetal OR fetus OR embryo* OR ((pregnan* OR first OR second OR third OR 1st OR 2nd OR 3rd OR 1-st OR 2-nd OR 3-rd) ADJ3 trimester*)).ab,ti.) NOT (exp animals/ NOT humans/) AND english.la.

**Google scholar (200 top-ranked)**

sequencing|sequence intitle:guideline|intitle:guidelines prenatal|prenatally|"pre-natal"|foetal|foetus|fetal|fetus|embryo|embryonal|pregnancy

| **Database searched** | **via** | **Years of coverage** | **Records** | **Records after duplicates removed** |
| --- | --- | --- | --- | --- |
| Embase | Embase.com | 1971 - Present | 1334 | 1315 |
| Medline ALL | Ovid | 1946 - Present | 565 | 143 |
| Other sources: Google Scholar (200 top-ranked) | | | 200 | 176 |
| **Total** | | | **2099** | **1634** |

**Manual search of the following professional societies:**

The American College of Medical Genetics (ACMG)

The American College of Obstetricians and Gynecologists (ACOG)

The Canadian College of Medical Genetics (CCMG)

The European Society for Human Genetics (ESHG)

The International Society for Prenatal Diagnosis (ISPD)

The International Society of Ultrasound in Obstetrics and Gynecology (ISUOG)

The Society for Maternal-Fetal Medicine (SMFM)

The Society of Obstetricians and Gynaecologists of Canada (SOGC)

The Perinatal Quality Foundation (PQF)

The National Society of Genetic Counselors (NSGC)

The British Society for Genetic Medicine (BSGM)

**Supplement 2**

Extended descriptions of the uncertainties

**a. Diagnostic yield**

More pregnant couples can receive results explaining their child’s fetal anomalies. Two recent studies have independently estimated prenatal Exome Sequencing (ES) to come to a diagnostic or potentially clinically relevant variant in 12.5% (of 610 fetuses)^1^ and 10% (of 234 fetuses)^2^ of unselected cases when karyotype or CMA results were negative. Thus, it is not self-evident that ES will provide a diagnosis.^3,4^

**b. Gene-disease correlations**

Uncertainty surrounding gene-disease correlations, refers to the limited information available on the postnatal and prenatal phenotype associated with a genetic variant. It is therefore unclear how a variant will affect a child during prenatal or postnatal development and inconclusive whether this variant is the cause of the structural anomaly.

**c. How a genetic anomaly presents prenatally**

Unique to the prenatal setting, uncertainty exists about how a genetic anomaly with a well-known postnatal phenotype presents prenatally. One of the major challenges at this moment is that most of the genotype-phenotype associations that we know of have been established after birth. Our knowledge of prenatal genotype-phenotype associations is limited.^5^ Therefore, comparing the genotype in the fetal period with the known postnatal phenotype is in some cases not possible, making it more difficult to come to a conclusive diagnosis prenatally.^6-9^

**d. Pathogenicity and variants of unknown significance (VUS)**

ES findings may be of uncertain pathogenicity, i.e. Variants of Unknown Significance (VUS or VOUS), which do not confirm or exclude diagnosis.^10^

**e. Secondary findings**

ES findings may be unrelated to the clinical phenotype. Another anomaly or syndrome can be found in the fetus that does not match the phenotype or indication for diagnosis.^11^ If these are actively looked for (e.g. part of the list of 59 medically actionable genes comprised by the ACMG for the postnatal setting),^12^ these are described as secondary findings.

**f. Incidental findings**

Findings that are unexpected or found by chance are referred to as incidental findings . Incidental findings also encompass results that might have consequences for parents or other family members. For example, in trio analysis, DNA of the parents is used to filter out irrelevant variants. This is beneficial for rapid analysis and can be beneficial for the diagnostic yield, but it also opens the possibility of finding e.g. conditions in the parents or non-paternity.^7,13^

**g. Technical validity of a result**

The technical validity of a result is an uncertainty for the healthcare professional. These may involve false positives, false negatives or an insufficient read-depth or no coverage, or difficult to detect low-level mosaicism to have certainty about a result. In these cases, further analyses and interpretation may be required.^14^ However, prenatally there is not always enough time to conduct additional analyses.

**h. Possible incomplete result**

Additionally, there is the uncertainty accompanied by an incomplete result. For example, in case of a finding where one autosomal recessive variant is found to be compatible with the fetal phenotype, but no second variant is found. Uncertainty for the healthcare professional here may lie in whether to report this finding or not.

**i. Incomplete penetrance**

Uncertainty about penetrance pertains to the chance that a pathogenic variant presents with symptoms. The anomaly might lead to symptoms in some but not all cases.

**j. Variable expression variants**

In the case of variable expression penetrance is 100%, but it is unclear to what degree symptoms will present themselves. One person with the same variant will be affected only mildly, while another is affected severely.

**References**

1. Lord J, McMullan DJ, Eberhardt RY, et al. Prenatal exome sequencing analysis in fetal structural anomalies detected by ultrasonography (PAGE): a cohort study. *Lancet.* 2019;393(10173):747-757.

2. Petrovski S, Aggarwal V, Giordano JL, et al. Whole-exome sequencing in the evaluation of fetal structural anomalies: a prospective cohort study. *Lancet.* 2019;393(10173):758-767.

3. Berg JS, Khoury MJ, Evans JP. Deploying whole genome sequencing in clinical practice and public health: meeting the challenge one bin at a time. *Genet Med.* 2011;13(6):499-504.

4. Westerfield L, Darilek S, Van den Veyver IB. Counseling challenges with variants of uncertain significance and incidental findings in prenatal genetic screening and diagnosis. *J Clin Med.* 2014;3(3):1018-1032.

5. Mellis R, Chandler N, Chitty LS. Next-generation sequencing and the impact on prenatal diagnosis. *Expert Rev Mol Diagn.* 2018;18(8):689-699.

6. Bui TH, Raymond FL, Van den Veyver IB. Current controversies in prenatal diagnosis 2: should incidental findings arising from prenatal testing always be reported to patients? *Prenat Diagn.* 2014;34(1):12-17.

7. Best S, Wou K, Vora N, Van der Veyver IB, Wapner R, Chitty LS. Promises, pitfalls and practicalities of prenatal whole exome sequencing. *Prenat Diagn.* 2018;38(1):10.

8. Mackie FL, Carss KJ, Hillman SC, Hurles ME, Kilby MD. Exome sequencing in fetuses with structural malformations. *J Clin Med.* 2014;3(3):747-762.

9. Abou Tayoun AN, Spinner NB, Rehm HL, Green RC, Bianchi DW. Prenatal DNA sequencing: clinical, counseling, and diagnostic laboratory considerations. *Prenat Diagn.* 2018;38(1):26-32.

10. Wallis Y, Payne S, McAnulty C, et al. Practice guidelines for the evaluation of pathogenicity and the reporting of sequence variants in clinical molecular genetics. *Association for Clinical Genetic Science and the Dutch Society of Clinical Genetic Laboratory Specialists.* 2013.

11. Vears DF, Sénécal K, Clarke AJ, et al. Points to consider for laboratories reporting results from diagnostic genomic sequencing. *Eur J Hum Genet.* 2018;26(1):36-43.

12. Kalia SS, Adelman K, Bale SJ, et al. Recommendations for reporting of secondary findings in clinical exome and genome sequencing, 2016 update (ACMG SF v2. 0): a policy statement of the American College of Medical Genetics and Genomics. *Genet Med.* 2017;19(2):249-255.

13. Van den Veyver IB, Eng CM. Genome-wide sequencing for prenatal detection of fetal single-gene disorders. *Cold Spring Harb Perspect Med.* 2015;5(10):a023077.

14. Weiss MM, Van der Zwaag B, Jongbloed JDH, et al. Best practice guidelines for the use of Next‐Generation Sequencing applications in genome diagnostics: a national collaborative study of Dutch genome diagnostic laboratories. *Hum Mutat.* 2013;34(10):1313-1321.
